# Awareness of congenital cytomegalovirus and acceptance of maternal and newborn screening

**DOI:** 10.1371/journal.pone.0221725

**Published:** 2019-08-26

**Authors:** Katie J. Tastad, Mark R. Schleiss, Sara M. Lammert, Nicole E. Basta

**Affiliations:** 1 Division of Epidemiology and Community Health, School of Public Health, University of Minnesota, Minneapolis, MN, United States of America; 2 Division of Pediatric Infectious Diseases and Immunology, School of Medicine, University of Minnesota, Minneapolis, MN, United States of America; University of British Columbia, CANADA

## Abstract

**Objectives:**

To assess awareness of cytomegalovirus (CMV); attitudes towards screening; and frequency of behaviors that could increase the risk of prenatal infection.

**Methods:**

We conducted a survey among 726 women at the 2017 Minnesota State Fair. Minnesota residents aged 18–44 were eligible if they had never been pregnant or had been pregnant within the past 10 years. We compared responses between never-pregnant and recently-pregnant women.

**Results:**

Only 20% of study participants had previously heard of CMV. Remarkably, recently-pregnant women were no more likely to be aware of CMV than never-pregnant women after adjusting for potential confounders. After receiving information about CMV, nearly all participants indicated they believed prenatal (96%) or newborn (96%) screening should be offered.

**Conclusions:**

Although baseline awareness of CMV was low (even among recently-pregnant women), after learning more about the risks, women supported screening. Several states have passed or proposed legislation promoting CMV education and/or screening programs. We identified important gaps in knowledge about CMV among women who may benefit from education about how to reduce their risk of exposure and who may need to decide whether they would be willing to screen for CMV in the future.

## Introduction

Cytomegalovirus (CMV) is a commonly acquired infection and a member of the *Herpesviridae* family. Among healthy individuals, CMV infection usually causes no symptoms; however, when CMV is transmitted congenitally (congenital CMV or cCMV) it can cause severe sequelae. Each year approximately 0.7% of newborns in the United States are infected with CMV *in utero* [1,2]. Approximately 20% of congenital CMV infections result in permanent disabilities, including microcephaly, hearing loss, vision loss, cerebral palsy, seizure disorders, or cognitive impairment [2]. The prevalence of cCMV is higher than Down syndrome, fetal alcohol syndrome, and spina bifida [3]. CMV is typically transmitted via direct mucosal contact with infected bodily fluids such as urine or saliva. The risk of congenital transmission is greatest in the setting of a primary maternal infection in pregnancy. Evidence suggests that contact with the urine or saliva of young children may be an important source of infection among women of reproductive age, and avoiding such exposures is a target for risk reduction interventions that could be offered to women during pregnancy [4,5]. Recommendations regarding strategies for promoting avoidance of direct contact with saliva (for example, avoiding sharing food, cups or utensils), and to encourage handwashing after every contact with diapers, are generally well-accepted approaches [6,7] and can be effective for reducing prenatal CMV infection [4,8,9].

Despite the relatively high prevalence of cCMV infections and potentially serious effects, many women are unfamiliar with the risk of cCMV [5,6,10–12]. Prior studies have estimated that only 9% [12] to 22% [5] of women have heard of cCMV. Similarly, efforts have been made to compare cCMV awareness between parents and non-parents [12,13] or between ever- versus never-pregnant women [5]. However, to our knowledge no prior studies have compared cCMV awareness between recently pregnant women, who have likely had access to education about healthy pregnancies during prenatal care, and women who have never been pregnant. Such comparisons could enhance our understanding of whether women who recently experienced a pregnancy received adequate information about cCMV during, or since that time. In addition, only one study has evaluated women’s opinions about newborn screening for cCMV [14]. This nationally representative survey found that about 85% of parents would want to have their newborn tested for CMV. While recommendations tend to include efforts to reduce exposure to saliva and urine, few studies have assessed the frequency with which women report engaging in activities that could lead to exposure [6,11]. Understanding current knowledge, attitudes, or practices regarding cCMV is particularly important at present because multiple states are considering or implementing legislative initiatives to promote cCMV education and/or screening programs. As of 2018, ten states have passed legislation that mandate providing cCMV education, and five more states (including Minnesota) have proposed such legislation. Five states have mandates requiring cCMV screening (or the offer of screening) for newborns who fail the newborn hearing screening [15].

We conducted a survey among women who had recently been pregnant and women who had never been pregnant to assess: 1) knowledge of the risks of CMV infection during pregnancy; 2) attitudes towards CMV screening in pregnancy and cCMV screening as part of a newborn screening program; and 3) frequency of activities that may increase risk of CMV exposure. The aim of our study was to compare recently-pregnant women with never-pregnant women to assess the relative understanding of each group regarding the risks of cCMV. Such a comparison, in turn, could yield insights into the question of what information is being conveyed, either during routine prenatal care or via other sources.

## Methods

### Data collection

We conducted a survey at the University of Minnesota Driven to Discover (D2D) Research Facility [16] at the Minnesota State Fair between August 27–31, 2017. The Minnesota State Fair draws over 1.7 million attendees each year [17], and in 2017 over 60,000 fair attendees visited the D2D Research Facility [16]. Volunteer staff recruited participants by engaging with fairgoers at the D2D Facility, describing the survey and the incentive to participate. Interested individuals completed a screening form to determine if they met the following eligibility criteria: female; aged 18–44 years; Minnesota residents at least six months of the year; able to read and speak English fluently; not currently pregnant, and either had never been pregnant (never-pregnant women) or had been pregnant within the past 10 years (recently-pregnant women). The 10-year threshold was chosen to reflect a time period over which it would be reasonable for respondents to recall, during which we might expect somewhat similar prenatal care, and to balance the need to enroll a sufficiently large sample of previously pregnant women. Eligible potential participants reviewed consent information prior to answering survey questions. Participants received a University of Minnesota drawstring backpack containing fact sheets on cCMV. Our target sample size of 600 participants reflected our primary aim to estimate the proportion of participants who had heard about cCMV prior to the survey.

This study was reviewed and approved by the University of Minnesota Institutional Review Board (STUDY00003321).

A 10-minute survey was developed based on prior mailed surveys in HealthStyles [11] of knowledge, attitudes, and practices regarding cCMV. Eight women who met study inclusion criteria pilot tested the survey. The survey included questions about participant demographics, knowledge of cCMV and other diseases and conditions, attitudes towards screening for CMV and cCMV, and practices that are thought to be risk factors for CMV infection. First, we asked questions about awareness of cCMV and other diseases and conditions. In the list of diseases, we included one fictitious item, “jolivirus”, to assess internal validity of responses; particularly whether a high proportion of respondents would check all conditions to advance quickly through the survey. We performed additional sensitivity analyses of cCMV knowledge by calculating the percentage that had heard of cCMV and running adjusted logistic regression analyses excluding women who indicated they had heard of every disease and condition presented, including jolivirus. After participants indicated their baseline knowledge of cCMV, we provided concise educational information adapted from a Fact Sheet by the National CMV Foundation[18] including incidence and outcomes associated with cCMV. We then gathered data from participants regarding their screening preferences. Attitudes towards possible screening options were assessed using a five-point Likert scale, ranging from “strongly disagree” to “strongly agree”. To assess practices that might increase the risk of CMV transmission, we asked women six questions about behaviors that could potentially lead to the transmission of CMV and, as a control, seven questions about behaviors that are unlikely to increase the risk of CMV transmission for comparison. Four of the questions about behaviors that may increase the risk of CMV transmission were adapted from the HealthStyles survey [11]. Our survey instructed participants who had at least one biological child to answer the questions with that child in mind. Participants who did not have children were asked if they were to have a child, how often did they think they would engage in the activities described. The complete survey, including instructions and educational text, is provided in supplementary material online (S1 Table).

Participants completed the survey using Apple iPads, and responses were automatically recorded in a secure RedCap software database [19]. All survey questions required a response to continue through the survey except for questions relating to demographic information. If a participant entered a value outside of the accepted range for continuous responses such age or year, a message would appear requesting that they confirm their response. Participants could then choose to keep their original entry or revise it.

Study advertising materials did not reference cytomegalovirus or CMV by name. This strategy was intentional, and was meant to ensure that participants would not be more likely to state they had heard of CMV, or suspect it as the most common cause of birth defects, when presented with a list of diverse diseases and conditions.

### Statistical analysis

Data were analyzed using SAS 9.4 (Carey, NC) and Stata 14 (StataCorp LP, College Station, TX). Descriptive statistics included calculating means for continuous variables and proportions for categorical variables. Responses from recently-pregnant women were compared with responses from never-pregnant women using t-tests (for continuous responses) and chi-squared tests (for dichotomous responses) along with corresponding 95% confidence intervals. Questions regarding knowledge, attitudes, and practices were compared between the two strata (never-pregnant and recently-pregnant women) using multivariable logistic regression. Dependent variables based on multiple choice questions that asked participants to select the correct answer were coded as correct (1) or incorrect (0) to model the odds of identifying the correct response. Models were adjusted for potential confounding variables, which were selected *a priori* due to likely associations with the exposure and the outcome of interest [11]. We accounted for the potential confounding effects of the following demographic variables in our models by adjusting for: age (categorical; (18–24; 25–29; 30–34; 35–39; and 40–44 years), education (binary; bachelor’s degree or higher versus less than a bachelor’s degree (including “other”)), race/ethnicity (categorical; White, non-Hispanic; Asian, non-Hispanic; Other), income (categorical: annual income Under $20,000; $20,000–59,999; $60,000–99,999; or $100,000+) and history of working in a daycare (binary: Yes; No/I Don’t Know) or in healthcare (binary: Yes; No/I Don’t Know).

## Results

### Participants

Of 856 individuals who were screened, 743 were eligible and 733 completed the survey (98.7% participation rate among those eligible). Seven participants who completed the survey were excluded from analysis because their responses to age or pregnancy history questions in the survey deemed them ineligible, resulting in 726 responses included in our analysis.

### Demographics

The mean age of the 726 participants was 28.6 years. Over half of the respondents had a bachelor’s degree or higher (58.8%). Overall, 83.3% reported their race as white, and 95.7% were not Hispanic or Latino. Ninety percent were born in the United States (Table 1). Overall, 28.1% (n = 204) of women reported that they were recently-pregnant while 71.9% (n = 522) were never-pregnant. Twenty women reported that they had been pregnant, but that they had no biological children. Recently-pregnant women were older on average than never-pregnant women (35.4 years versus 25.9 years, respectively, (9.5 years difference 95% CI: 8.5–10.6), p <0.0001) although both groups included women from the full range of eligible ages (18–44 years). A greater percentage of recently-pregnant women than never-pregnant women held a Bachelor’s degree or higher (71.6% versus 58.3%, respectively, 13.3% difference, p = 0.0002) (Table 1).

**Table 1:** Demographics of the 726 participants who completed the survey by pregnancy history.

Category	Subcategory	All n = 726	Recently Pregnant n = 204	Never Pregnant n = 522
		n (%)	n (%)	n (%)
Age	Mean (Standard Deviation)	28.6 (7.7)	35.4 (5.8)	25.9 (6.6)
Highest Level of Education Attained	Some high school	9 (1.2)	1 (0.5)	8 (1.5)
	High school diploma or GED	70 (9.6)	8 (3.9)	62 (11.9)
	Some college, no degree	146 (20.1)	22 (10.8)	124 (23.8)
	Associate's degree	72 (9.9)	27 (13.2)	45 (8.6)
	Bachelor's degree	272 (37.5)	80 (39.2)	192 (36.8)
	Graduate or professional degree	155 (21.3)	66 (32.4)	89 (17.0)
	Other	2 (0.3)	0	2 (0.4)
Ethnicity	Hispanic or Latino	31 (4.3)	5 (2.5)	26 (5.0)
	Not Hispanic or Latino	695 (95.7)	199 (97.5)	496 (95.0)
Race	White	605 (83.3)	181 (88.7)	424 (81.2)
	Asian	64 (8.8)	12 (5.9)	52 (10.0)
	Multiracial	24 (3.3)	4 (2.0)	20 (3.8)
	American Indian or Alaska Native	11 (1.5)	2 (1.0)	9 (1.7)
	Other	11 (1.5)	1 (0.5)	10 (1.9)
	Black or African American	9 (1.2)	3 (1.5)	6 (1.1)
	Hawaiian or Other Pacific Islander	1 (0.1)	1 (0.5)	0
	Missing	1 (0.1)	0	1 (0.2)
US Born	No	67 (9.2)	13 (6.4)	54 (10.3)
	Yes	659 (90.8)	191 (93.6)	468 (89.7)
Household Income	Under $20,000	111 (15.3)	8 (3.9)	103 (19.7)
	$20,000–59,999	235 (32.4)	41 (20.1)	194 (37.2)
	$60,000–99,999	186 (25.6)	57 (27.9)	129 (24.7)
	$100,000+	192 (26.4)	98 (48.0)	94 (18.0)
	Missing	2 (0.3)	0	2 (0.4)
Worked in Daycare	Yes	165 (22.7)	48 (23.5)	117 (22.4)
	No	557 (76.7)	156 (76.5)	401 (76.8)
	I don't know	4 (0.6)	0	4 (0.8)
Worked as Healthcare Provider	Yes	220 (30.3)	80 (39.2)	140 (26.8)
	No	500 (68.9)	121 (59.3)	379 (72.6)
	I don't know	6 (0.8)	3 (1.5)	3 (0.6)
Number of Biological Children	0	20 (2.8)	20 (9.8)	0
	1	46 (6.3)	46 (22.5)	0
	2	76 (10.5)	76 (37.3)	0
	3	45 (6.2)	45 (22.1)	0
	4	13 (1.8)	13 (6.4)	0
	5	2 (0.3)	2 (1.0)	0
	6	2 (0.3)	2 (1.0)	0
Plan to Become Pregnant in Future	Yes	391 (53.9)	54 (26.5)	337 (64.6)
	No	210 (28.9)	125 (61.3)	85 (16.3)
	I don't know	125 (17.2)	25 (12.3)	100 (19.2)
When Planning to Become Pregnant *(Among those planning to become pregnant in the future)*	Within 1 year	40 (10.2)	20 (37.0)	20 (5.9)
	In 1–5 years	193 (49.4)	32 (59.3)	161 (47.8)
	In 6–10 years	123 (31.5)	1 (1.9)	122 (36.2)
	In more than 10 years	18 (4.6)	0	18 (5.3)
	I don't know	17 (4.3)	1 (1.9)	16 (4.7)

### Knowledge, attitudes, and practices

**Fig 1:**
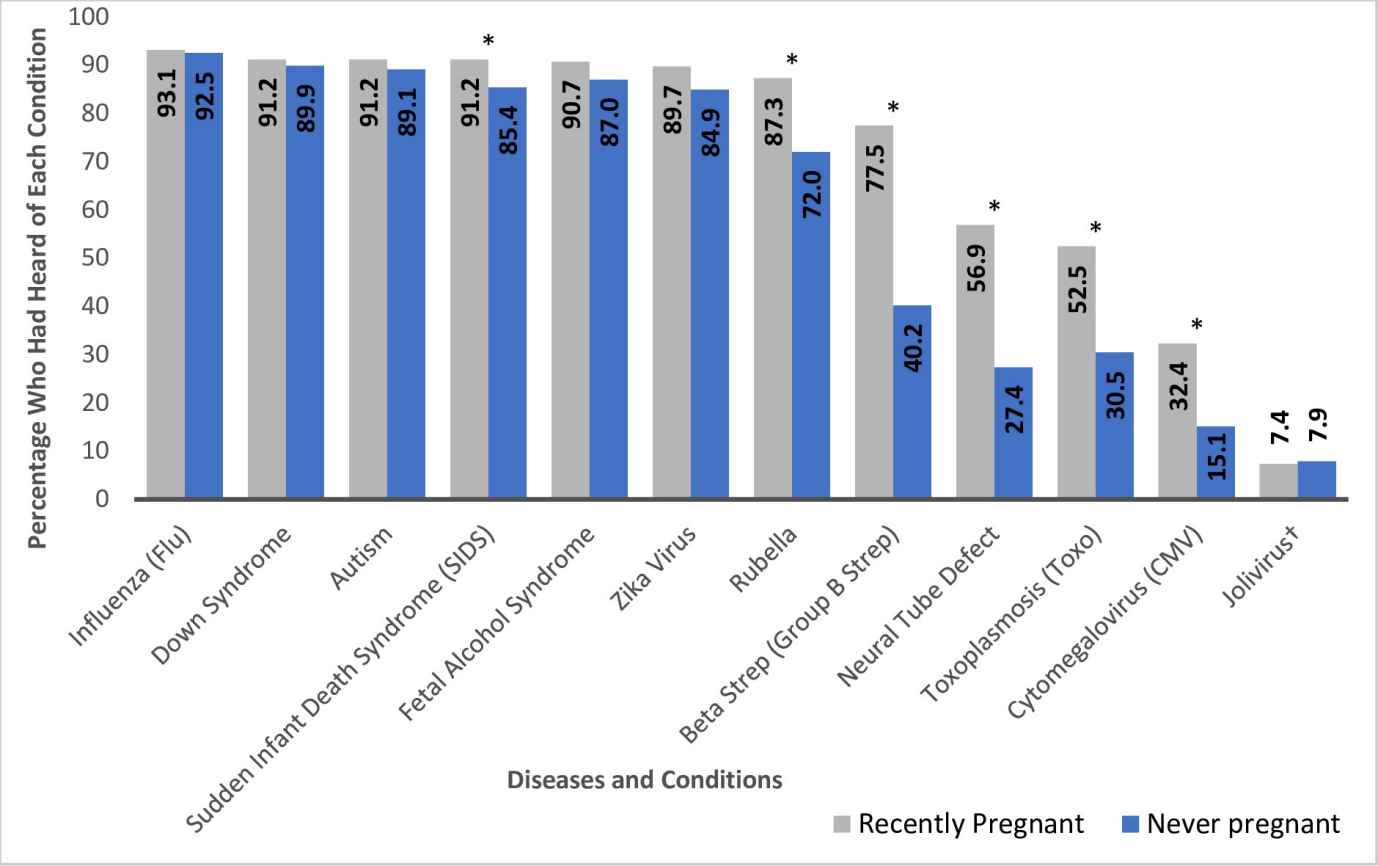
Awareness of diseases and conditions by pregnancy history. * Statistically significant differences between those recently-pregnant (grey bars) and those never-pregnant (blue bars) (chi-square test p-value < 0.05). ^+^ Jolivirus is a fictitious virus included to assess validity of survey responses.

Only 20% of participants indicated that they had heard of cCMV–indeed, fewer had heard of cCMV than any of the other 10 conditions listed. Most women were familiar with influenza, Down syndrome, autism, sudden infant death syndrome (SIDS), and fetal alcohol syndrome (Fig 1). As part of our data quality check, we noted that 7.7% indicated that they had heard of the fictitious “jolivirus”, and that 3.0% of participants had selected all 12 conditions. Among the women who did not identify the fictitious jolivirus, regardless of their other responses, 17.9%. (120/670) indicated they were aware of CMV. Among the women who had not chosen every response including jolivirus, 17.5% (123/704) had indicated that they were aware of cCMV.

Among all women with knowledge of cCMV, the most commonly cited sources of information were school (37.9%) and work (36.6%). When answering multiple choice questions about cCMV, only 13.1% of all participants correctly responded that cCMV was the most common cause of birth defects; and only 14.9% correctly identified that hearing loss was the most common problem associated with cCMV. Over half of women (55.0%) correctly responded that screening for CMV was not typically offered during pregnancy in the United States, though a quarter were not sure if it was. Fewer women correctly responded that newborns are not usually screened for cCMV in the United States (38.6%). A complete description of the distribution of responses is provided in Table 2 and S1 Table.

**Table 2:** Knowledge of congenital cytomegalovirus (CMV) and prenatal and newborn CMV screening by pregnancy status comparing recently pregnant with never pregnant women (OR >1 indicates greater odds among recently-pregnant than among never-pregnant women).

	Total (n = 726)		Recently pregnant (n = 204)		Never Pregnant (n = 522)		Unadjusted Model (Recently versus Never Pregnant)	Adjusted Model^a^ (Recently versus Never Pregnant)	
	n	%	n	%	n	%	OR (95% CI)	OR (95% CI)	p-value^f^
Heard of CMV	145	20.0	66	32.4	79	15.1	2.7 (1.8, 3.9)	1.5 (0.9, 2.6)	0.12
Aware that CMV is the Most Common Cause of Birth Defects ^b^	95	13.1	27	13.2	68	13.0	1.0 (0.6, 1.6)	0.8 (0.4, 1.4)	0.44
Aware that Hearing Loss is Most Common Problem among infants with cCMV^c^	108	14.9	42	20.6	66	12.6	1.8 (1.2, 2.7)	1.4 (0.8, 2.4)	0.19
Aware that Pregnant women are not usually screened for CMV ^d^	399	55.0	119	58.3	280	53.6	1.2 (0.9, 1.7)	1.2 (0.8, 1.8)	0.37
Aware that Newborns not usually screened for CMV ^e^	280	38.6	94	46.1	186	35.6	1.5 (1.1, 2.1)	1.3 (0.8, 1.9)	0.25

^a^Adjusted for age category, education, race/ethnicity, income, prior/current work in healthcare, and prior/current work in daycare (n = 724).

^b^ Modeled the odds of correctly selecting “Congenital Cytomegalovirus (CMV)” when asked “Which of the following do you think causes the most birth defects among babies born in the United States?”

^c^ Modeled the odds of correctly selecting “Hearing Loss” when asked “What do you think the most common problem babies with congenital cytomegalovirus have?”

^d^ Modeled the odds of correctly answering “No” versus “Yes” or “I don’t Know” when asked “Do you think women are usually screened for CMV during pregnancy in the United States?”

^e^ Modeled the odds of correctly answering “No” versus “Yes” or “I don’t Know” when asked “Do you think newborns are usually screened for CMV in the United States?”

^f^ P-value for recently-pregnant versus never-pregnant comparison.

After learning more about the incidence and risks of CMV and cCMV, 96.4% of women believed that screening should be offered to pregnant women, and 76.6% indicated they would choose prenatal screening for themselves if they were to become pregnant in the future. Similarly, 96.2% believed that newborn screening should be offered, and 82.1% would choose to have their baby to be screened for cCMV if they were to have a baby in the future (Table 3).

**Table 3:** Attitudes towards prenatal and newborn cytomegalovirus screening by pregnancy history.

		All (n = 726)	Recently Pregnant (n = 204)	Never Pregnant (n = 522)	Chi-Square p-value
Question	Response	n (%)	n (%)	n (%)	
**Prenatal Screening for CMV**					
Think it should be offered	Strongly agree	368 (50.7)	81 (39.7)	287 (55.0)	<0.0001
	Somewhat agree	147 (20.2)	48 (23.5)	99 (19.0)	
	Agree	185 (25.5)	59 (28.9)	126 (24.1)	
	Somewhat disagree	20 (2.8)	12 (5.9)	8 (1.5)	
	Strongly disagree	6 (0.8)	4 (2.0)	2 (0.4)	
Would choose to be screened	Yes	556 (76.6)	132 (64.7)	424 (81.2)	<0.0001
	No	56 (7.7)	29 (14.2)	27 (5.2)	
	I don't know	114 (15.7)	43 (21.1)	71 (13.6)	
**Newborn Screening for CMV**					
Think it should be offered	Strongly agree	404 (55.6)	93 (45.6)	311 (59.6)	0.0062
	Somewhat agree	125 (17.2)	47 (23.0)	78 (14.9)	
	Agree	169 (23.3)	52 (25.5)	117 (22.4)	
	Somewhat disagree	21 (2.9)	9 (4.4)	12 (2.3)	
	Strongly disagree	7 (1.0)	3 (1.5)	4 (0.8)	
Would choose for baby to be screened	Yes	596 (82.1)	149 (73.0)	447 (85.6)	0.0005
	No	38 (5.2)	17 (8.3)	21 (4.0)	
	I don't know	91 (12.5)	37 (18.1)	54 (10.3)	
	Missing	1 (0.1)	1 (0.5)	0	

When asked about behavioral practices, a high proportion of women reported engaging in activities that may increase risk of CMV transmission, including kissing their child on the lips (80.7%), sharing a cup (83.2%), eating utensils (82.2%), or food (88.6%) with their child “rarely” or “often”. As expected, nearly all women responded they would participate in common care-taking behaviors with their child, including bathing the child (97.4%), and changing diapers (98.3%) (Table 4).

**Table 4:** Frequency of common behaviors/practice among parents of young children, some of which could lead to transmission of pathogens and may contribute to child-to-mother CMV transmission (marked with an ^+^).

	Respondent Group	Frequency of behavior/practice			
	Had biological child(ren) ^a^	Often	Rarely	Never	Chi-Square p-value
**Behavior/Practice**	** **	n (%)	n (%)	n (%)	** **
Kiss child on lips^+^	Yes	138 (75.0)	28 (15.2)	18 (9.8)	< .0001
	No	224 (41.3)	196 (36.2)	122 (22.5)	
Share cup^+^	Yes	119 (64.7)	54 (29.3)	11 (6.0)	< .0001
	No	223 (41.1)	208 (38.4)	111 (20.5)	
Share utensils^+^	Yes	110 (59.8%)	62 (33.7%)	12 (6.5%)	< .0001
	No	236 (43.5%)	189 (34.9%)	117 (21.6%)	
Share food^+^	Yes	145 (78.8%)	35 (19.0%)	4 (2.2%)	< .0001
	No	296 (54.6%)	167 (30.8%)	79 (14.6%)	
Clean pacifier with mouth^+^	Yes	62 (33.7%)	45 (24.5%)	77 (41.8%)	< .0001
	No	91 (16.8%)	130 (24.0%)	321 (59.2%)	
Share toothbrush^+^	Yes	25 (13.6%)	21 (11.4%)	138 (75.0%)	0.0686
	No	45 (8.3%)	82 (15.1%)	415 (76.6%)	
Give child a bath	Yes	178 (96.7)	6 (3.3)	0	0.027
	No	499 (92.1)	24 (4.4)	19 (3.5)	
Wash child's hair	Yes	175 (95.1)	9 (4.9)	0 (0.0)	0.0007
	No	459 (84.7)	65 (12.0)	18 (3.3)	
Change diaper	Yes	184 (100)	0	0	0.0086
	No	515 (95.0)	15 (2.8)	12 (2.2)	
Kiss child on cheek or top of head	Yes	183 (99.5)	1 (0.5)	0	0.0002
	No	489 (90.2)	36 (6.6)	17 (3.1)	
Give child pacifier	Yes	130 (70.7%)	32 (17.4%)	22 (12.0%)	0.0141
	No	410 (75.6%)	102 (18.8%)	30 (5.5%)	
Clean pacifier	Yes	127 (69.0%)	33 (17.9%)	24 (13.0%)	< .0001
	No	456 (84.1%)	53 (9.8%)	33 (6.1%)	
Brush teeth	Yes	173 (94.0%)	10 (5.4%)	1 (0.5%)	0.0007
	No	451 (83.2%)	63 (11.6%)	28 (5.2%)	

^a^ Women with one or more biological children were asked to answer based on when their youngest child was in diapers. Women who had no biological children were asked to answer what they thought they would do if they were to care for a child who was still in diapers.

### Differences in knowledge, attitudes, and practices between groups

Recently-pregnant women were more likely to have heard of *Streptococcus agalactiae* (Group B Strep), cCMV, Neural Tube Defect, Rubella, Sudden Infant Death Syndrome (SIDS), and Toxoplasmosis (Toxo) than never-pregnant women (chi-square tests of proportions p<0.05) (Fig 1). Higher education, older age group, and history of working in healthcare were all positively associated with cCMV awareness. After adjusting for these factors, there was no significant difference in cCMV awareness by pregnancy history in the logistic regression model, or in sensitivity analysis that excluded participants who indicated they had heard of all conditions listed, including jolivirus (results not shown).

Never-pregnant women were more likely to strongly agree that newborn screening should be offered (59.6% compared with 45.6% of recently-pregnant women, chi-square p-value <0.001). Never-pregnant women were also more likely to state they would choose newborn screening if given the option in the future; 85.6% would choose newborn screening compared with 73.0% of recently-pregnant women (chi-square, p-value <0.001) (Table 3). About 90% of women who had children responded that they had kissed their young child on the lips and about 58% had cleaned a pacifier with her own mouth (Table 4). Women without children expected to perform these behaviors less frequently, with 77% indicating they would kiss their child on the lips, and 41% stating they would clean a pacifier with their own mouth (chi-square p-values for both comparisons <0.001) (Table 4).

## Discussion

We found that awareness of cCMV among women aged 18–44 years was low regardless of pregnancy history, with only 20% of all women of reproductive age indicating awareness of the infection prior to the survey. The majority of participants incorrectly assumed cCMV was less common than other, more widely discussed causes of pregnancy complications and congenital syndromes. Participants (especially recently-pregnant participants) reported frequently engaging in common behaviors that could increase risk for child-to-parent transmission of CMV such as sharing food, cups, or utensils. Because infants and young children with CMV infection typically demonstrate sustained, high-level shedding of virus in urine and saliva [20], activities that could expose pregnant women to viral shedding an potentially transmit the pathogen are of concern. Our results demonstrate a need for greater education and awareness among women of reproductive age about the risks of CMV transmission and cCMV. Our results also indicate that once women become aware of CMV, they demonstrate interest in potential engagement in screening programs, both for themselves and for their newborn infants. As never-pregnant women were more strongly in favor of screening programs than recently-pregnant women, this could indicate that a woman’s past experience of prenatal care or parenthood impacts her attitudes towards screening.

We designed our survey to assess issues related to cCMV awareness and attitudes toward screening using a more detailed and comprehensive approach than other surveys. This allowed us to evaluate multiple aspects of individual awareness, attitudes, and preferences regarding cCMV. However, comparing our results to other surveys can elucidate trends. For example, the nationally-representative HealthStyles survey found decreases in cCMV awareness among women ranging from 14% in 2005, to 13% in 2010, and only 9% in both 2015 and 2016 [12]. At 20%, our 2017 survey indicated a higher percentage of awareness among our sample group (which included a high proportion of highly educated women in Minnesota) than the HealthStyles surveys. High acceptability of newborn screening in our study (82% stated they would want their newborn tested) was consistent with the 2009 HealthStyles survey that found 85% of parents would want their newborn tested even if it were not routine, if they had to pay $20, or if CMV-related problems never developed in their baby [14].

While we conducted a comprehensive survey among a large number of respondents to learn more about cCMV awareness and attitudes towards screening, our survey is limited in that we drew a convenience sample from Minnesota State Fair attendees choosing to visit the University of Minnesota D2D Research Facility, who are unlikely to be representative of the general population. Among all survey participants, 59% had a bachelor’s degree or higher, compared with 40% of all Minnesotans aged 25–44 [21]. In previous studies, higher educational attainment was associated with greater knowledge about cCMV [11,12]. Our survey sample included a high proportion of highly educated respondents and, as a result, our estimates of the proportion of women knowledgeable about cCMV may actually overestimate awareness compared to a random sample of the general population. Due to the limited participation of individuals from a diversity of backgrounds, we were not able to assess how awareness may differ based on race, ethnicity, or other factors. Future studies should seek greater participation of individuals from underrepresented racial and ethnic groups. Self-reported responses can be subject to recall and response biases. To reduce recall bias relating to pregnancy or early child-care, we included only women who had been pregnant within the past 10 years, rather than “ever-pregnant”, in the “recently-pregnant” group. We minimized the potential for participants to give perceived desirable responses by designing a self-administered survey. In addition, we included several measures to assess internal validity. We included “jolivirus” in the list of diseases and conditions, to assess whether a high proportion of respondents would check all conditions to advance through the survey more quickly, but we did not find cause for concern. Participants received only basic information about cCMV and CMV screening in our study and may not have fully understood reasons why screening might not be desirable (e.g. the experience of a false positive screen). Our study aimed to assess hypothetical support for screening generally, consistently with previous studies. Future studies should provide comprehensive information to participants to ensure they are able to critically evaluate the potential benefits and harms of prenatal or newborn screening. and to assess their likely real-world decisions. In the list of behaviors, we included numerous common behaviors, including both practices that could increase the risk of CMV, and practices that have not been shown to increase risk of CMV transmission (e.g. giving a child a bath), to avoid the appearance that “never” or “rarely” were the preferred responses.

Overall, our study provides a large, robust assessment of current cCMV awareness and attitudes among women of reproductive age, a key target group for increasing cCMV awareness. Legislation regarding CMV was first proposed in Minnesota in 2018, and would have mandated that education about the risks of CMV be provided to both the general public and to healthcare providers [22]. Our study provides data about baseline awareness that can help inform policy decisions and can be used to evaluate the population-level impact of new policies in the future. The question of whether women should be routinely screened for CMV antibodies before and/or during pregnancy is controversial and warrants further research [23]. However, increasing knowledge and awareness of cCMV among women of child-bearing age could have an impact on the risk of acquisition of infection [4]. Strategies to reduce acquisition of CMV during pregnancy are needed, since prevention of maternal infection could reduce adverse neurodevelopmental and audiological outcomes in children. This study identifies key areas that can be targeted to increase awareness of and education about cCMV and to reduce the potential risk of CMV transmission.

## Conclusions

Our findings indicate that awareness of cCMV remains low among women of reproductive age, and surprisingly in our study population women who had completed a pregnancy had the same low level of knowledge of CMV as women who had never been pregnant. We also demonstrate that after learning basic information about the risks of cCMV, women are interested in both prenatal and newborn screening. We have identified important gaps in knowledge about cCMV among women of reproductive age. These women may both benefit from education about cCMV, and may, if provided with the opportunity, choose to screen themselves or their newborns for CMV infection. These findings are timely, as several states have passed or proposed legislation mandating cCMV education and/or screening. Increasing cCMV knowledge and awareness among women has the potential to reduce risks of CMV exposure during pregnancy. However, more information is needed to determine how best to provide education and support for woman who would like to reduce their risk of CMV, and about what strategies can put into place that will help identify newborns who may benefit from early detection of cCMV infection.

## Supporting information

S1 Table Full survey results for all participants and by pregnancy history. (DOCX)
