# Supplementary material for: Awareness of congenital cytomegalovirus and acceptance of maternal and newborn screening
Source: PLoS One. 2019 Aug 26;14(8):e0221725. doi: 10.1371/journal.pone.0221725 (PMC6709948; doi:10.1371/journal.pone.0221725)
Supplement: S1 Table — (DOCX) [file pone.0221725.s001.docx]

**Supplemental Appendix 1: Full survey results for all participants and by pregnancy history**

| **Question** | **Response options for categorical variables** | **All**  **(n=726)** | **Recently Pregnant (n=204)** | **Never**  **Pregnant**  **(n=522)** |
| --- | --- | --- | --- | --- |
|  |  | n (%) | n (%) | n (%) |
| Please enter your age. |  | 28.6 (7.7)* | 35.4 (5.8)* | 25.9 (6.6)* |
| What is the highest level of education you have attained? | Some high school | 9 (1.2) | 1 (0.5) | 8 (1.5) |
|  | High school diploma or GED | 70 (9.6) | 8 (3.9) | 62 (11.9) |
|  | Associate’s degree | 72 (9.9) | 27 (13.2) | 45 (8.6) |
|  | Some college, no degree | 146 (20.1) | 22 (10.8) | 124 (23.8) |
|  | Bachelor’s degree | 272 (37.5) | 80 (39.2) | 192 (36.8) |
|  | Graduate or professional degree | 155 (21.3) | 66 (32.4) | 89 (17.0) |
|  | Other | 2 (0.3) | 0 (0.0) | 2 (0.4) |
| What is your ethnicity? | Hispanic or Latino | 31 (4.3) | 5 (2.5) | 26 (5.0) |
|  | Not Hispanic or Latino | 695 (95.7) | 199 (97.5) | 496 (95.0) |
| What is your racial background? | American Indian or Alaska Native | 11 (1.5) | 2 (1.0) | 9 (1.7) |
|  | Asian | 64 (8.8) | 12 (5.9) | 52 (10.0) |
|  | Black or African American | 9 (1.2) | 3 (1.5) | 6 (1.1) |
|  | Hawaiian or Other Pacific Islander | 1 (0.1) | 1 (0.5) | 0 (0.0) |
|  | White | 605 (83.3) | 181 (88.7) | 424 (81.2) |
|  | Multiracial | 24 (3.3) | 4 (2.0) | 20 (3.8) |
|  | Other | 11 (1.5) | 1 (0.5) | 10 (1.9) |
|  | Missing | 1 (0.1) | 0 (0.0) | 1 (0.2) |
| Were you born in the United States? | Yes | 659 (90.8) | 191 (93.6) | 468 (89.7) |
|  | No | 67 (9.2) | 13 (6.4) | 54 (10.3) |
| Please enter your ZIP code. |  |  |  |  |
| Have you ever worked in a daycare facility (either in a group daycare, or with daycare in your or someone else’s home)? | Yes | 165 (22.7) | 48 (23.5) | 117 (22.4) |
|  | No | 557 (76.7) | 156 (76.5) | 401 (76.8) |
|  | I don’t know | 4 (0.6) | 0 (0) | 4 (0.8) |
| Have you ever worked as a healthcare professional (as a physician, nurse, or in another patient-care occupation)? | Yes | 220 (30.3) | 80 (39.2) | 140 (26.8) |
|  | No | 500 (68.9) | 121 (59.3) | 379 (72.6) |
|  | I don’t know | 6 (0.8) | 3 (1.5) | 3 (0.6) |
| What is your household income? | Under $20,000 | 111 (15.3) | 8 (3.9) | 103 (19.7) |
|  | $20,000-59,999 | 235 (32.4) | 41 (20.1) | 194 (37.2) |
|  | $60,000-99,999 | 186 (25.6) | 57 (27.9) | 129 (24.7) |
|  | $100,000+ | 192 (26.4) | 98 (48.0) | 94 (18.0) |
|  | Missing | 2 (0.3) | 0 (0) | 2 (0.4) |
| Before today had you ever heard of the following diseases/conditions? (mark “Yes” or “No” for each disease/condition) | Autism | 651 (89.7) | 186 (91.2) | 465 (89.1) |
|  | Beta Strep (Group B Strep) | 368 (50.7) | 158 (77.5) | 210 (40.2) |
|  | Cytomegalovirus (CMV) | 145 (20.0) | 66 (32.4) | 79 (15.1) |
|  | Down Syndrome | 655 (90.2) | 186 (91.2) | 469 (89.8) |
|  | Fetal Alcohol Syndrome | 639 (88.0) | 185 (90.7) | 454 (87.0) |
|  | Influenza (Flu) | 673 (92.7) | 190 (93.1) | 483 (92.5) |
|  | Jolivirus | 56 (7.7) | 15 (7.4) | 41 (7.9) |
|  | Neural Tube Defect | 259 (35.7) | 116 (56.9) | 143 (27.4) |
|  | Rubella | 554 (76.3) | 178 (87.3) | 376 (72.0) |
|  | Sudden Infant Death Syndrome (SIDS) | 632 (87.1) | 186 (91.2) | 446 (85.4) |
|  | Toxoplasmosis (Toxo) | 266 (36.6) | 107 (52.5) | 159 (30.5) |
|  | Zika Virus | 626 (86.2) | 183 (89.7) | 443 (84.9) |
| Which of the following do you think causes the most birth defects among babies born in the United States? | Congenital Cytomegalovirus (CMV) | 95 (13.1) | 27 (13.2) | 68 (13.0) |
|  | Down Syndrome | 122 (16.8) | 36 (17.6) | 86 (16.5) |
|  | Fetal Alcohol Syndrome | 417 (57.4) | 113 (55.4) | 304 (58.2) |
|  | Jolivirus | 6 (0.8) | 1 (0.5) | 5 (1.0) |
|  | Neural Tube Defect | 64 (8.8) | 21 (10.3) | 43 (8.2) |
|  | Zika Virus | 22 (3.0) | 6 (2.9) | 16 (3.1) |
| Interesting Info: Congenital CMV is the most common congenital infection in the United States – it’s effects are more common than Down Syndrome, Fetal Alcohol Syndrome, Neural Tube Defects, and Zika.  If you are wondering about Jolivirus, that one isn’t a real threat (but the name sure sounds catchy)! | | | | |
| Where have you heard about Cytomegalovirus (CMV)? (select all that apply) | Friend or family member | 37 (5.1) | 12 (5.9) | 25 (4.8) |
|  | Healthcare provider | 57 (7.9) | 34 (16.7) | 23 (4.4) |
|  | Internet or other media | 56 (7.7) | 23 (11.3) | 33 (6.3) |
|  | School | 76 (10.5) | 22 (10.8) | 54 (10.3) |
|  | Someone I know was affected by CMV | 12 (1.7) | 4 (2.0) | 8 (1.5) |
|  | Work | 63 (8.7) | 24 (11.8) | 39 (7.5) |
|  | This survey is the first time I have heard of CMV | 464 (63.9) | 110 (53.9) | 354 (67.8) |
|  | Other | 49 (6.7) | 15 (7.4) | 34 (6.5) |
| What do you think is the most common problem babies with congenital Cytomegalovirus (CMV) have? | Cataracts | 18 (2.5) | 4 (2.0) | 14 (2.7) |
|  | Cleft lip | 153 (21.1) | 35 (17.2) | 118 (22.6) |
|  | Hearing loss | 108 (14.9) | 42 (20.6) | 66 (12.6) |
|  | Heart defect | 323 (44.5) | 91 (44.6) | 232 (44.4) |
|  | Intestinal defects | 124 (17.1) | 32 (15.7) | 92 (17.6) |
| Interesting info: The most common problem caused by congenital Cytomegalovirus (CMV) is hearing loss. Babies can also have microcephaly (small head size), developmental disabilities, vision loss, and cerebral palsy.  In adults, CMV is a common infection - over half of people in the United States will have CMV by age 40. Once a person has CMV, it stays for life. It is usually harmless, and many will never know they were infected. | | | | |
| Just under 70,000 babies are born in Minnesota every year. About how many babies do you think are born with congenital CMV in Minnesota each year? |  | 8416.1 (46831.4)* | 13896.1  (85720.4)* | 6274.5  (13135.7)* |
| Interesting info: An estimated 700 babies are born in Minnesota with congenital CMV every year (about 0.5-1). Risk for congenital CMV is highest when a mother is infected with CMV for the first time while pregnant. | | | | |
| Pregnant women undergo many screenings while pregnant. Do you think women are usually screened for CMV during pregnancy in the United States? | Yes | 142 (19.6) | 40 (19.6) | 102 (19.5) |
|  | No | 399 (55.0) | 119 (58.3) | 280 (53.6) |
|  | I don’t know | 185 (25.5) | 45 (22.1) | 140 (26.8) |
| Newborn babies undergo many screenings shortly after being born. Do you think newborns are usually screened for CMV in the United States? | Yes | 293 (40.4) | 74 (36.3) | 219 (42.0) |
|  | No | 280 (38.6) | 94 (46.1) | 186 (35.6) |
|  | I don't know | 153 (21.1) | 36 (17.6) | 117 (22.4) |
| Interesting info:   - Most of the time, a woman who is infected with CMV will not pass the virus on to her baby (up to 40 will pass it on). - Most babies who are born with CMV are healthy (about 10 have problems at birth, and an additional 10-20 develop hearing problems). - Babies affected by congenital CMV can benefit from early interventions for hearing loss and developmental delay.   While CMV is a common infection, problems due to congenital CMV infection are relatively rare. | | | | |
| Screening during pregnancy can determine if a woman is infected or has been infected with CMV sometime in the past. How strongly do you agree or disagree that pregnant women should be offered screening for CMV? | Strongly agree | 368 (50.7) | 81 (39.7) | 287 (55.0) |
|  | Somewhat agree | 147 (20.2) | 48 (23.5) | 99 (19.0) |
|  | Agree | 185 (25.5) | 59 (28.9) | 126 (24.1) |
|  | Somewhat disagree | 20 (2.8) | 12 (5.9) | 8 (1.5) |
|  | Strongly disagree | 6 (0.8) | 4 (2.0) | 2 (0.4) |
| If you were to become pregnant in the future, do you think you would choose to be screened for CMV if it were offered to you? | Yes | 556 (76.6) | 132 (64.7) | 424 (81.2) |
|  | No | 56 (7.7) | 29 (14.2) | 27 (5.2) |
|  | I don’t know | 114 (15.7) | 43 (21.1) | 71 (13.6) |
| Screening can determine if a newborn has congenital CMV even if he or she has no symptoms. How strongly do you agree or disagree that parents should be offered CMV screening for their newborn babies? | Strongly agree | 404 (55.6) | 93 (45.6) | 311 (59.6) |
|  | Somewhat agree | 125 (17.2) | 47 (23.0) | 78 (14.9) |
|  | Agree | 169 (23.3) | 52 (25.5) | 117 (22.4) |
|  | Somewhat disagree | 21 (2.9) | 9 (4.4) | 12 (2.3) |
|  | Strongly disagree | 7 (1.0) | 3 (1.5) | 4 (0.8) |
| If you were to have a baby in the future, would you want your baby to be screened for CMV? | Yes | 596 (82.1) | 149 (73.0) | 447 (85.6) |
|  | No | 38 (5.2) | 17 (8.3) | 21 (4.0) |
|  | I don’t know | 91 (12.5) | 37 (18.1) | 54 (10.3) |
|  | Missing | 1 (0.1) | 1 (0.5) | 0 (0.0) |
| Interesting info: Currently in the United States, screening for CMV is not routinely recommended during pregnancy or for newborns. | | | | |
| Have you ever been pregnant? | Yes | 204 (28.1) | 204 (100.0) |  |
|  | No | 522 (71.9) | 522 (100.0) |  |
| How many biological children do you have?  *(Only women indicating “Yes” to “Have you ever been pregnant” answered this question.)* | 0 | 20 (2.8) | 20 (9.8) |  |
|  | 1 | 46 (6.3) | 46 (22.5) |  |
|  | 2 | 76 (10.5) | 76 (37.3) |  |
|  | 3 | 45 (6.2) | 45 (22.1) |  |
|  | 4 | 13 (1.8) | 13 (6.4) |  |
|  | 5 | 2 (0.3) | 2 (1.0) |  |
|  | 6 | 2 (0.3) | 2 (1.0) |  |
| *[For women indicating they had at least 1 biological child]:* When your youngest child was in diapers, how often did you personally do each of the following?  *If you have more than 1 biological child, please answer the following questions with your youngest biological child in mind.*  -OR-  [*For women indicating either they had never been pregnant, or had been pregnant and had 0 biological children]:*  If you had a child and you cared for the child while the child was still in diapers, how often do you think would you personally do the following? | | | | |
| Give the child a bath | Often | 677 (93.3) | 196 (96.1) | 481 (92.1) |
|  | Rarely | 30 (4.1) | 7 (3.4) | 23 (4.4) |
|  | Never | 18 (2.5) | 0 (0.0) | 18 (3.4) |
| Wash the child’s hair | Often | 634 (87.3) | 192 (94.1) | 442 (84.7) |
|  | Rarely | 74 (10.2) | 12 (5.9) | 62 (11.9) |
|  | Never | 18 (2.5) | 0 (0.0) | 18 (3.4) |
| Change the child’s diaper | Often | 699 (96.3) | 203 (99.5) | 496 (95.0) |
|  | Rarely | 15 (2.1) | 1 (0.5) | 14 (2.7) |
|  | Never | 12 (1.7) | 0 (0.0) | 12 (2.3) |
| Kiss the child on the cheek or top of the head | Often | 672 (92.6) | 201 (98.5) | 471 (90.2) |
|  | Rarely | 37 (5.1) | 2 (1.0) | 35 (6.7) |
|  | Never | 17 (2.3) | 1 (0.5) | 16 (3.1) |
| Kiss the child on the lips | Often | 362 (49.9) | 150 (73.5) | 212 (40.6) |
|  | Rarely | 224 (30.9) | 33 (16.2) | 191 (36.6) |
|  | Never | 140 (19.3) | 21 (10.3) | 119 (22.8) |
| Share a cup with the child | Often | 342 (47.1) | 128 (62.7) | 214 (41.0) |
|  | Rarely | 262 (36.1) | 64 (31.4) | 198 (37.9) |
|  | Never | 122 (16.8) | 12 (5.9) | 110 (21.1) |
| Share eating utensils with the child | Often | 346 (47.7) | 121 (59.3) | 225 (43.1) |
|  | Rarely | 251 (34.6) | 69 (33.8) | 182 (34.9) |
|  | Never | 129 (17.8) | 14 (6.9) | 115 (22.0) |
| Share food (take bites from the same food) with the child | Often | 441 (60.7) | 155 (76.0) | 286 (54.8) |
|  | Rarely | 202 (27.8) | 45 (22.1) | 157 (30.1) |
|  | Never | 83 (11.4) | 4 (2.0) | 79 (15.0) |
| Give the child a pacifier | Often | 540 (74.4) | 145 (71.1) | 395 (75.7) |
|  | Rarely | 134 (18.5) | 36 (17.6) | 98 (18.8) |
|  | Never | 52 (7.2) | 23 (11.3) | 29 (5.6) |
| Clean a pacifier with water and soap | Often | 583 (80.3) | 145 (71.1) | 438 (83.9) |
|  | Rarely | 86 (11.8) | 34 (16.7) | 52 (10.0) |
|  | Never | 57 (7.9) | 25 (12.3) | 32 (6.1) |
| Clean a pacifier with your mouth | Often | 153 (21.1) | 69 (33.8) | 84 (16.1) |
|  | Rarely | 175 (24.1) | 51 (25.0) | 124 (23.8) |
|  | Never | 398 (54.8) | 84 (41.2) | 314 (60.2) |
| Brush the child’s teeth | Often | 624 (86.0) | 191 (93.6) | 433 (83.0) |
|  | Rarely | 73 (10.1) | 12 (5.9) | 61 (11.7) |
|  | Never | 29 (4.0) | 1 (0.5) | 28 (5.4) |
| Share a toothbrush with the child | Often | 70 (9.6) | 29 (14.2) | 41 (7.9) |
|  | Rarely | 103 (14.2) | 24 (11.8) | 79 (15.1) |
|  | Never | 553 (76.2) | 151 (74.0) | 402 (77.0) |
| When your youngest child was in diapers, did he or she regularly attend a daycare center outside the home (not including an in-home daycare provider) for a month or more?) | Yes | 97 (13.4) | 97 (47.5) | 0 (0.0) |
|  | No | 85 (11.7) | 85 (41.7) | 0 (0.0) |
|  | I don’t know | 2 (0.3) | 2 (1.0) | 0 (0.0) |
|  | Missing | 542 (74.7) | 20 (9.8) | 522 (100) |
| Do you plan to become pregnant in the future? | Yes | 391 (53.9) | 54 (26.5) | 337 (64.6) |
|  | No | 210 (28.9) | 125 (61.3) | 85 (16.3) |
|  | I don’t know | 125 (17.2) | 25 (12.3) | 100 (19.2) |
| How soon do you intend to become pregnant?  *(Only women indicating “Yes” they plan to become pregnant in the future answered this question.)* | Within 1 year | 40 (10.2) | 20 (37.0) | 20 (5.9) |
|  | In 1-5 years | 193 (49.4) | 32 (59.3) | 161 (47.8) |
|  | In 6-10 years | 123 (31.5) | 1 (1.9) | 122 (36.2) |
|  | In more than 10 years | 18 (4.6) | 0 (0.0) | 18 (5.3) |
|  | I don’t know | 17 (4.3) | 1 (1.9) | 16 (4.7) |
| While congenital CMV is the most common cause of congenital problems, it is still relatively rare in the US. There are steps you can take to prevent yourself from contracting CMV and other germs, including:  Washing hands with soap and water – especially after changing diapers, feeding young children  wiping a child’s nose, and handling children’s toys  Not sharing food, cups, or eating utensils with young children  Not putting a child’s pacifier in your own mouth  Not sharing a toothbrush with a young child  Avoiding contact with saliva when kissing a young child  Cleaning all toys, countertops and other surfaces that have come into contact with urine or saliva. | | | | |

* Standard deviation reported rather than percent for continuous variables.
